# Differential *var* gene expression in the organs of patients dying of falciparum malaria

**DOI:** 10.1111/j.1365-2958.2007.05837.x

**Published:** 2007-08

**Authors:** Jacqui Montgomery, Fingani A Mphande, Matthew Berriman, Arnab Pain, Stephen J Rogerson, Terrie E Taylor, Malcolm E Molyneux, Alister Craig

**Affiliations:** 1 Malawi-Liverpool-Wellcome Programme of Clinical Tropical Research, College of Medicine Blantyre, Malawi; 2 Liverpool School of Tropical Medicine Liverpool, UK; 3 The Pathogen Sequencing Unit, Wellcome Trust Sanger Institute Hinxton, UK; 4 Department of Medicine, The University of Melbourne Parkville, Australia; 5 Blantyre Malaria Project Blantyre, Malawi; 6 College of Osteopathic Medicine, Michigan State University East Lansing, MI, USA

## Abstract

Sequestration of parasitized erythrocytes in the microcirculation of tissues is thought to be important in the pathogenesis of severe falciparum malaria. A major variant surface antigen, *var*/*Plasmodium falciparum* erythrocyte membrane protein 1, expressed on the surface of the infected erythrocyte, mediates cytoadherence to vascular endothelium. To address the question of tissue-specific accumulation of variant types, we used the unique resource generated by the clinicopathological study of fatal paediatric malaria in Blantyre, Malawi, to analyse *var* gene transcription in patients dying with falciparum malaria. Despite up to 102 different *var* genes being expressed by *P. falciparum* populations in a single host, only one to two of these genes were expressed at high levels in the brains and hearts of these patients. These major *var* types differed between organs. However, identical *var* types were expressed in the brains of multiple patients from a single malaria season. These results provide the first evidence of organ-specific accumulation of *P. falciparum* variant types and suggest that parasitized erythrocytes can exhibit preferential binding in the body, supporting the hypothesis of cytoadherence-linked pathogenesis.

## Introduction

The capacity of *Plasmodium falciparum* to cause severe and fatal disease is believed to be in part due to its ability to sequester in post-capillary venules. The process of cytoadherence is mediated by a variety of host endothelial receptors and by *P. falciparum* antigens expressed on the surface of the host erythrocyte. The best studied of these is the *P. falciparum* erythrocyte membrane protein 1 (PfEMP1), encoded by the *var* multigene family (Baruch *et al*., 1995). Each mature asexual parasite expresses a dominant PfEMP1 type that can be stably inherited through successive cell cycles or can switch to expression of a different gene (Scherf *et al*., 1998).

PfEMP1 proteins are composed of adhesive domains, termed Duffy-binding-like (DBL), constant (C2) and cysteine-rich interdomain region. These domains can be sorted into subgroups by sequence motifs and are characterized by distinctive binding properties such that specific domains determine to which endothelial receptors each *P. falciparum*-infected erythrocyte (pRBC) adheres (Buffet *et al*., 1999; Smith *et al*., 2000). pRBC expressing PfEMP1 with defined binding specificities can be selected from a mixed population by adhesion to particular endothelial receptors.

*var* genes have been classified into various groups (A–E) based on coding and non-coding sequence motifs and domain arrangements. Most genes contain a DBLα domain at their N-terminus which can be further subgrouped into α (sometimes labelled DBLα_0_) and α_1_ types that contain two or four conserved cysteines residues respectively (Robinson *et al*., 2003). DBLα_1_ domains are characteristic of type A and B/A *var* genes that do not adhere to CD36 but some of which can mediate rosetting, the binding of pRBC to uninfected erythrocytes (Rowe *et al*., 1997; Russell *et al*., 2005). Type A *var* genes have been associated with severe or cerebral in peripheral populations although such studies have produced conflicting results (Kirchgatter and del Portillo, 2002; Jensen *et al*., 2004; Kaestli *et al*., 2004; 2006; Bull *et al*., 2005; Kyriacou *et al*., 2006; Rottmann *et al*., 2006).

Genetically variant isolates of *P. falciparum* contain overlapping but generally distinct contingents of *var* genes and sequence relatedness is independent of geographic origin and strain type, apart from some areas of low transmission (Kyes *et al*., 1997; Albrecht *et al*., 2006; Barry *et al*., 2007). A few unusual *var*, such as the *var2csa* gene implicated in malaria in pregnancy, are highly conserved in different *P. falciparum* populations (Salanti *et al*., 2002; Trimnell *et al*., 2006; Kraemer *et al*., 2007). *P. falciparum* in monoclonal infections express single or a few dominant *var* genes in circulating populations (Peters *et al*., 2002; Bull *et al*., 2005; Lavstsen *et al*., 2005; Kyriacou *et al*., 2006). A longitudinal study of asymptomatic hosts demonstrated that *var* gene expression changes dramatically over time, with minimal overlap in *var* repertoire between samples taken at 2 week intervals despite few changes in *P. falciparum* genetic types in the infecting population (Kaestli *et al*., 2004).

One of the limitations of these previous studies is that it has only been possible to study the dynamics of *var* expression in the peripheral blood. We have been conducting a clinicopathological study of fatal paediatric malaria in Blantyre, Malawi, since 1996 (Taylor *et al*., 2004). Using this resource, we recently analysed the distribution of pRBC in the peripheral blood, five sites in the brain and seven other organs by genotyping the merozoite surface protein 1 and 2 (*msp*1/2) alleles, a commonly used technique for identifying genetically distinct *P. falciparum* types (Snounou *et al*., 1999; Farnert *et al*., 2001). Types amplified from the peripheral blood tended to be detected throughout the body but infections in the organs were more complex than in the peripheral blood. We compared infections in fatal cerebral malaria (CM) patients with parasitaemic children who had non-malarial causes of death (Montgomery *et al*., 2006). Relative to parasitaemic controls, CM patients had less complex infections, and genetic types were distributed more homogenously throughout the organs. *msp* type was not associated with the site of sequestration.

We have now examined *var* gene expression by *P. falciparum* parasites in the brain, lung, heart and spleen of fatal paediatric malaria patients. Because of the extremely complex nature of the expression of this gene family, a small number of patients were studied in detail. We found dominant expression of particular *var* genes within a tissue population, and that the dominant form varied between organs. Some of these dominant *var* types were detected in the same organs of other patients from the same malaria season. This finding provides preliminary evidence that the repertoire of *var* genes mediating organ-specific sequestration within a season may be limited.

## Results and discussion

### Pilot study

We analysed the diversity of *var* transcripts expressed by parasites in the organs of six cases of fatal falciparum malaria. RNA was extracted from the brain, lung, heart and spleen, cDNA was synthesized and DBL1α sequence amplified, cloned and sequenced. The samples consisted primarily of human genetic material and, for this reason, a single polymerase chain reaction (PCR) did not consistently amplify *P. falciparum* nucleic acid and so a previously described nested PCR was utilized (Duffy *et al*., 2002). These primers were previously optimized for minimal bias towards individual DBL1α sequences and bias was calculated in that study at less than 2.5% (Duffy *et al*., 2002). *var* sequences were considered identical when 99–100% similar, which corresponds to a maximum of 4 bp changes over the 340–450 bp sequence.

Twenty *var* clones were sequenced from each organ. There was a high degree of diversity, with up to 14 different *var* sequences identified from a single organ. Cloning was repeated from the tissues of one patient and the frequencies of individual *var* types were compared between the two reactions. While mainly the same transcripts were detected in both reactions, the proportions in each organ differed widely. We concluded that the number of clones examined was inadequate to study the observed level of diversity in *var* gene expression. The study design was altered to examine a larger number of transcripts in a subset of these patients to ensure a comprehensive analysis and to reduce potential PCR bias.

### Complex *var* expression in the organs of fatal malaria patients

Three cases of fatal paediatric malaria were chosen for the present study: PM30, who died of severe malarial anaemia (SMA) in the absence of coma; PM32, with a diagnosis of CM and SMA; and PM55, with CM alone (Table 1). DBL1α sequences were amplified from the brain, heart, lung and spleen of the three patients in two separate PCR reactions for each sample. The first three organs were chosen as they are major sites of *P. falciparum* sequestration. Unfortunately, peripheral blood samples were not collected from these patients at the time of autopsy. We chose to also analyse the spleen, in which pRBC are eliminated from the circulation or ‘pitted’ (removal of parasite without erythrocyte destruction; Angus *et al*., 1997; Chotivanich *et al*., 2002). pRBC found within the spleen may therefore be representative of the circulating population.

**Table 1:** Clinical details of patients.

	Diagnosis	Age (months)	Time to death (h : min)	Admission parasitaemia (parasites μl^−1^)	Final parasitaemia (parasites μl^−1^)	Date of admission
PM30	SMA	7	01:40	302 400	302 400	February 1999
PM32	CM + SMA	18	02:40	572 880	572 880	March 1999
PM55	CM	52	23:00	286 650	97 306	March 2001
Additional patients from pilot study						
PM36	CM + SMA	21	11:00	11 399	6350	April 1999
PM39	CM	18	17:40	6 030	100	May 1999
PM78	CM	15	02:00	637 000	637 000	May 2003

**Fig. 1:**
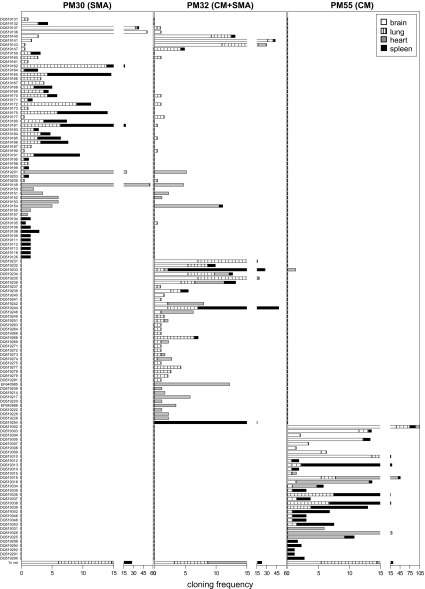
Frequency of *var* types in the organs of paediatric malaria patients. Each graph represents one patient (labelled above with final diagnosis in parentheses) with organs denoted by shading. *var* types are listed on the *y*-axis by accession number and the frequency of cloning is adjusted for the number of complete sequences analysed from each organ. Single copy *var* types are represented together and labelled 1× var.

Ninety-six products were cloned from each reaction, sequenced and aligned, with comparable frequencies of individual *var* types observed in the duplicate reactions. Sequences from the pilot study were also included in analyses. A total of 133–202 clones were obtained from each organ sample with 2020 clones overall. Hereafter, ‘clone’ will refer to each sequenced RT-PCR product, and ‘type’ will refer to each different DBL1α sequence identified. A median of 26 (range 11–49) different *var* types were amplified from each organ of the three patients. Figure 1 illustrates the cloning frequency of individual *var* types and the overlap between organs and patients. Half of the 248 *var* types were detected a single time in one organ only, accounting for only 6% of clones examined. All other *var* types were cloned more than once from a single organ or were detected in multiple organs and/or cases. The single copy clones were grouped together in Fig. 1 (1× var) and excluded from motif analysis.

The distribution of mature pRBC and *P. falciparum msp*1/2 genetic types has been previously described (Montgomery *et al*., 2006). There was no correlation between either of these factors and the diversity of *var* types detected in the organs of these patients. The homogeneous distribution of *msp* genetic types throughout the organs of CM patients is in contrast to the current findings, where up to 102 different *var* types are expressed by *P. falciparum* parasites in a single patient but only one to two types are expressed at high levels in brain and heart microvasculature.

The infections consisted of both mature and ring stage pRBC; however, immature asexual parasites have been shown to transcribe the same dominant *var* transcript as the mature stages that express PfEMP1 protein (Peters *et al*., 2002). Mature pRBC were not observed in the lungs, heart and spleen of PM55, although they were present at high number in the brain (Montgomery *et al*., 2006). Dense accumulations of pigment in these organs provided evidence of recent sequestered populations. However, this child had a high circulating parasitaemia at the time of death and the *var* expression observed here is assumed to be from immature stages transiently present in organs other than the brain, where their expression would be overwhelmed by the sequestered population.

### A subset of *var* types are expressed at high levels in the brain and heart

The number of *var* types amplified in each organ varied, with less diversity in the brain and heart (6.7 and 5.0 types/genotype respectively) than in the lung (8.4 types/genotype) and spleen (9.2 types/genotype). In most samples, one to two *var* types were detected at far higher frequency than other types in the same organ (Fig. 1 and Fig. S1). This was particularly obvious in all three brain samples, where the dominant types made up a third to a half of all clones detected in this tissue but were present in other organs only at low levels. These findings suggest organ-specific sequestration of particular *var* types and support the hypothesis that PfEMP1 type determines the site of cytoadherence.

Many of the less abundant *var* clones were detected in multiple organs within a patient; between 16% and 65% (median 47%) of *var* types detected in one tissue were detected in one or more other organs of the same patient. When the frequency at which each clone was detected was considered, the median overlap in detection of *var* types shifted to 77% (range 37–93%). There were no clones detected in all four organs of PM30. Four to six per cent of the *var* types amplified in PM32 and PM55 were detected in all organs, or up to 40% of clones. These types may represent pRBC sequestered in multiple organs, or *var* expression by circulating forms.

Major overlap in expression of *var* types between the lung and spleen was common in all three patients, accounting for 90% of lung and 80% of spleen clones in PM55, presumably mainly expressed by non-sequestered parasites. In PM32, 58% of brain *var* types were also detected in the lung, which accounted for 90% of all clones detected in the brain but only 57% of those from the lung. Despite these overlaps, the dominance in expression of particular *var* types in the brain and other organs strongly suggests that particular *var*/PfEMP1 types mediate sequestration in these tissues.

### Organ-specific *var* expression is observed in multiple patients from a single malaria season

We investigated if any *var* types were shared between the three patients. This was not expected as previous studies have shown minimal overlap in the expressed *var* repertoire between patients (Kaestli *et al*., 2004; Bull *et al*., 2005; Kyriacou *et al*., 2006). There was only one *var* type shared between PM55 and PM32, detected at two copies in the heart of PM55 and in all four organs of PM32. There were no *var* types shared between PM30 and PM55. Surprisingly, there was substantial overlap in the *var* types detected in PM30 and PM32, with 20 DBLα/*var* types detected in both cases (Fig. 1). These sequences were identical between the two patients. It is important to note that shared DBLα sequence does not necessarily imply that that entire *var* genes represented by each tag are identical. However, we will continue to refer to these as ‘*var* types’ for continuity.

The shared *var* types accounted for 20% of PM30 *var* types and 26% of PM32 *var* types, compromising 61% and 32% of all clones detected in each patient respectively. This overlap was particularly striking in the brain, with 26% of PM30 brain *var* types (90% of clones) also detected in the brain of PM32, including all of the dominant types in both cases. There was also major overlap in the heart of both patients, accounting for 42% of all PM30 *var* types in the heart (89% of clones) but only 14% of PM32 heart *var* types (24% of clones).

Why there should be such overlap in the *var* types expressed by parasites in these two infections is intriguing. PM30 and PM32 were hospitalized a month apart and were from widely separated villages, whereas PM55 was admitted to the study 2 years later. Data from the pilot study showed that two of the most highly transcribed *var* types in the brain of PM32, DQ519140 and DQ519141, also expressed in PM30, were additionally detected in the brain of a third child from the same season, PM36. This child was from the same district as PM30 but was admitted to the ward 2 months later (Table 1). None of these infections were comprised of similar *msp*1 and 2 genetic types (Montgomery *et al*., 2006). *var* expression from the infection of PM39, a fourth child from this season who was admitted 6 weeks subsequent to PM36, did not display any overlap in *var* types detected in the brains of the other cases. However, 15% of *var* types from the additional three cases in the pilot study were also detected in other patients, including each other and the three patients from the main study. The distribution of these shared types did not exhibit any tissue tropism.

As striking as the overlap in *var* types between patients is the fact that the organ localization is conserved. The potential of PfEMP1 as a vaccine candidate has been questionable due to its antigenically variant nature, although there is evidence that the *var* repertoire may be more restricted than originally thought (Kraemer and Smith, 2003; Bull *et al*., 2005). Using this molecule as a vaccine in malaria in pregnancy appears to be more promising in view of the very restricted and conserved nature of the PfEMP1 types involved in placental sequestration (Duffy *et al*., 2005). However, our finding that not only are a limited number of *var* types expressed in a dominant fashion in the brain (and heart) of malaria patients, but also that these types are shared between patients, suggests that some form of organ-specific *var* expression such as seen in placental malaria may also occur in severe paediatric malaria.

The patients described in this study carried genetically complex *P. falciparum* infections with up to seven genotypes detected in a single organ (Montgomery *et al*., 2006). We have shown here that these parasites expressed up to 49 *var* types in a single organ, and between 68 and 102 *var* types in an individual patient. It is possible that had we amplified more clones, we would have detected additional minor *var* types, although the dominance of particular types in some organs was evident (Fig. S1). That parasites can express over 100 *var* types in a single host, but only one to two types at high frequency in the brain, suggests that particular *var* genes are responsible for adherence in this organ.

It must be questioned whether the dominance of particular *var* types within an organ truly reflects differential expression levels, or whether this is due to biased amplification or cloning, or to contamination of circulating stages. For either of these to be true, we would expect that the same bias or contamination would be observed in all samples within a patient and therefore we would not see different *var* expression profiles between organ populations. There is disagreement regarding *var* expression in early asexual stages, with some researchers finding relaxed transcription of *var* types in early stages shifting to expression of a single *var* transcript in late asexual stages, and another study finding relaxed transcription even in late stages (Chen *et al*., 1998; Scherf *et al*., 1998; Kyes *et al*., 2000; Duffy *et al*., 2002). A recent paper demonstrated that the placental malaria-related gene, *var2csa*, appears to be the only *var* transcript expressed throughout the asexual life cycle (Schieck *et al*., 2007). However, as this is an atypical *var* gene, it is unclear how much this data can be extrapolated to the entire multigene family. Taken as a whole, the fact that the dominant *var* types observed in this study vary between organ populations argues strongly that they are not an artefact of bias or contamination by early asexual stages.

### DBL sequence motifs are not associated with organ localization

A general phylogenetic analysis of the *var* sequences did not display clustering associated with their detection in any of the four host organs (J. Montgomery, unpublished). Most of our sequences contained four cysteines with only 12% of *var* types (excluding those detected a single time) containing only two cysteine residues, a motif previously associated with severe malarial disease in infants (Kirchgatter and del Portillo, 2002). None of the major transcripts in any organ were of the two cysteine/DBLα_1_ sequence group except in the spleen of PM32 (DQ519244).

The sequences were then classified according to previously identified DBLα motifs such as the number of cysteine residues and positions of limited variance (PoLV; Bull *et al.* 2005). We have previously shown that the distribution of PoLV groups among these Malawian *var* types correspond well to other populations in Africa, Asia and South America (Bull *et al*., 2007). We now investigated whether *var* types consisting of particular PoLV groups were differentially distributed between tissues.

**Fig. 2:**
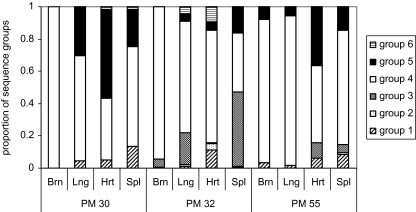
Frequency of sequence groups in *var* transcripts from the organs of paediatric malaria patients. Shading represents the sequence groups as identified by Bull *et al*. (2005), which are characterized by the number of cysteine residues and other semi-conserved motifs known as positions of limited variance. The data are expressed as the percentage of *var* types within each organ containing the corresponding sequence motifs. Brn, brain; Lng, lung; Hrt, heart; Spl, spleen.

Figure 2 shows the distribution of PoLV groups in our *var* sequences. None of the groups shows an association with the site of sequestration, even when adjusted for the frequency of cloning (data not shown). Expression levels of PoLV group 1 sequences were found to be negatively associated with the variant antibody repertoire in *P. falciparum*-infected Kenyan children (Bull *et al*., 2005). These sequences were found at low frequency in our data; immune regulation may preclude the expression of *var* types containing these conserved sequences in paediatric infections in areas of high malaria transmission such as Malawi.

### *var* sequences expressed in the hearts of two Malawian patients are similar to 3D7 *var* genes

Many of the *var* types expressed by *P. falciparum* in heart tissue of PM30 and PM32 were highly similar or identical to 3D7 *var* types. All of the 11 *var* types detected in the heart of PM30 displayed greater than 70% identity with *var* sequences from the 3D7 genome, and eight were greater than 80% identical to these genes. In pRBC from the heart of PM32, seven of 25 multiple copy *var* types displayed high similarity to 3D7 *var* types, plus three of the 13 single copy *var* types. With one exception (DQ519236), these 3D7-similar *var* types were exclusively expressed in the heart of PM32. The number of 3D7 *var* genes to which the Malawian isolates showed similarity was limited, with 24 Malawian DBL1α sequences displaying varying levels of similarity to 14 3D7 *var* genes.

### Conclusions

The expression of *var* genes in the human host is complex; half of the 248 *var* types were amplified a single time from a single organ, 29% were observed in multiple organs from the same patient, and 9% were detected in two patients. In the brain, at least, there is clear dominance of certain *var* types and the dominant types vary between organs. The data obtained in this study suggest that the PfEMP1 proteins encoded by only a small number of *var* genes are responsible for sequestration in brain microvasculature. An additional and intriguing finding is that there appears to be overlap between the *var* types expressed in the brains of children who have died of malaria within a single season. This finding suggests that the number of *var* types mediating sequestration in the brain may be limited, and if so, therapies capable of blocking or reversing adhesion of *P. falciparum* parasites in the brain may be feasible.

## Experimental procedures

### Clinical

Clinical details, including diagnosis and treatment, have been previously described (Montgomery *et al*., 2006). An initial pilot study examined four patients from the 1999 malaria season and one patient each from the 2001 and 2003 seasons. The selection of these cases was based on an autopsy-confirmed diagnosis of fatal malaria and a high peripheral parasitaemia at admission. The three patients examined in detail were chosen by high RNA yields from organ samples and their clinical details are outlined in Table 1.

This study was approved by ethics committees at the University of Malawi, Michigan State University and the University of Liverpool.

### RNA extraction

Organ samples collected at autopsy were snap frozen in liquid nitrogen in frozen tissue matrix (OCT compound, Tissue-Tek) and stored at −80°C. Approximately 0.5 g of frozen material was ground in liquid nitrogen and transferred to 10× volume of Trizol (Invitrogen), prewarmed to 37°C. Insoluble material was removed by centrifugation at 12 000 *g* for 10 min followed by incubation at room temperature for 5 min. Extraction was then performed according to manufacturer's instructions. RNA was treated for DNA contamination using a DNA-free RNA kit (Genetix) and complete removal was tested by PCR of DBL1α sequence as described below.

### cDNA synthesis, amplification and cloning

cDNA was synthesized from 2 μl of RNA using the Retroscript kit (Ambion) and quality was checked by agarose gel electrophoresis. One microlitre of cDNA initiated a primary PCR of DBL1α sequence using previously described oligonucleotides DBL-fo and DBL-ro (Duffy *et al*., 2002) at 1 μM final concentration and 1 mM dNTPs, 4 mM Mg^2+^ and 0.025 U *Taq* DNA polymerase (Qiagen). One microlitre of primary product was used for nested PCR with the same reaction components, oligonucleotides DBL-fi and DBL-ri and reaction conditions as described (Duffy *et al*., 2002). Products were purified using a QIAquick PCR purification or gel extraction kit (Qiagen) as required.

PCR products were ligated into the pGEM-T Easy vector (Promega) and transformed into *Escherichia coli* DH5α bacteria. Colonies were grown in liquid media and frozen in 96 well plates. Plasmid purification and DNA sequencing were performed at the Wellcome Trust Sanger Institute.

### Sequence analysis

Sequences were aligned using clustalw (http://align.genome.jp). Searches for sequence identity with the *P. falciparum* 3D7 genome were performed using *PlasmoDB* (http://www.plasmodb.org). DBL motifs were analysed using a database kindly provided by Peter Bull. These sequence data have been submitted to the DDBJ/EMBL/GenBank databases under accession numbers DQ519104–DQ519354, EF640985 and EF640986.

## Supplementary material

The following supplementary material is available for this article:

Fig. S1 Frequency of cloning of individual var types within the organs of Malawian paediatric malaria patients PM30 (A-D), PM32 (E-H) and PM55 (I-L). var types are listed in order of decreasing frequency of cloning within each tissue and are identified by accession number. The dashed line indicates 33% frequency of cloning, the cut-off point for dominance of one to two var types.

This material is available as part of the online article from: http://www.blackwell-synergy.com/doi/abs/10.1111/j.1742-4658.2006.05837.x (This link will take you to the article abstract).
