# Supplementary material for: Differential var gene expression in the organs of patients dying of falciparum malaria
Source: Mol Microbiol. 2007 Aug;65(4):959–67. doi: 10.1111/j.1365-2958.2007.05837.x (PMC2170262; doi:10.1111/j.1365-2958.2007.05837.x)
Supplement: Fig. S1 — Frequency of cloning of individual var types within the organs of Malawian paediatric malaria patients PM30 (A-D), PM32 (E-H) and PM55 (I-L). var types are listed in order of decreasing frequency of cloning within each tissue and are identified by accession number. The dashed line indicates 33% frequency of cloning, the cut-off point for dominance of one to two var types. [file mmi0065-0959-SD1.pdf]

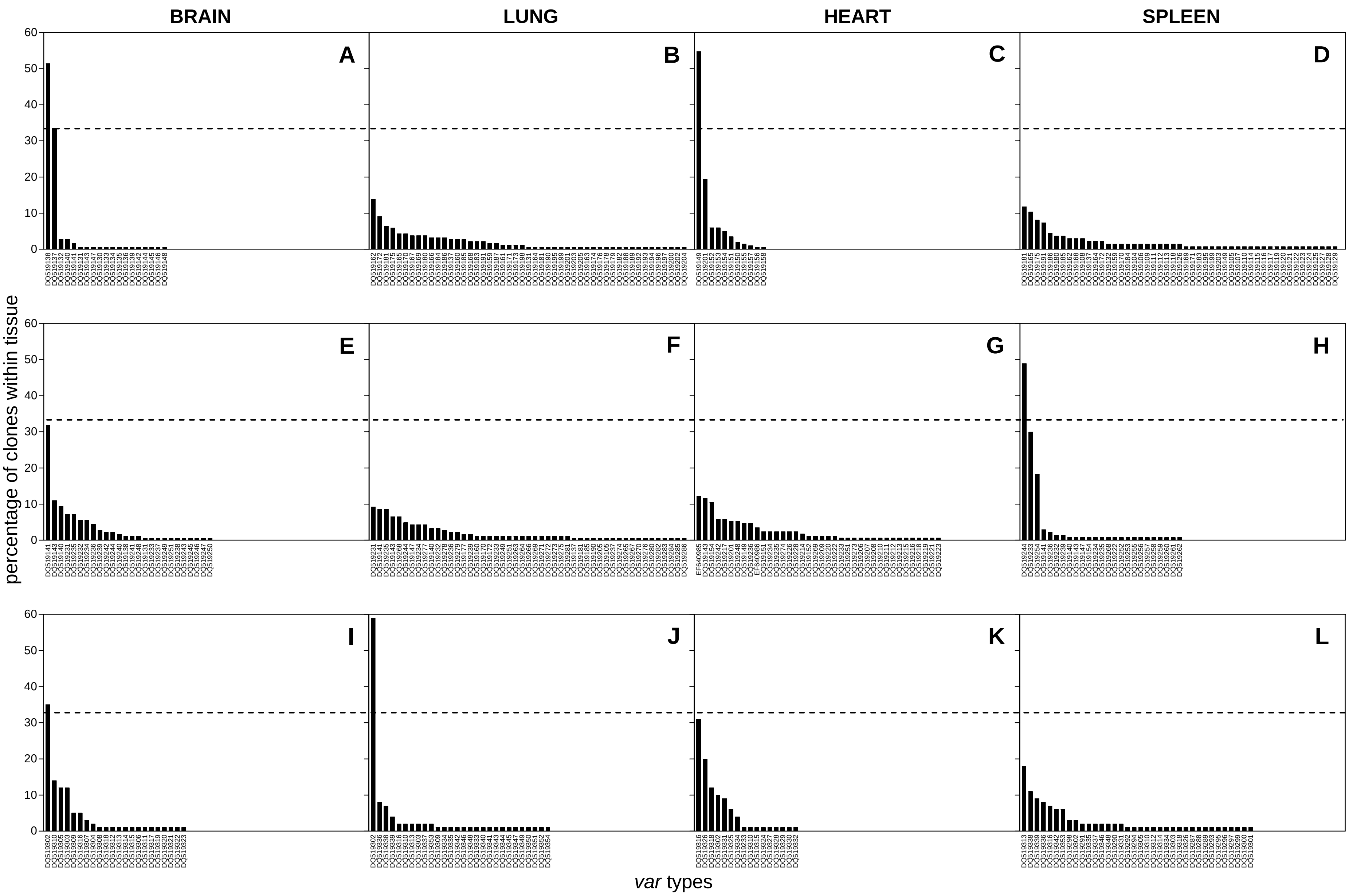
BRAIN
LUNG
HEART
SPLEEN
A
B
C
D
E
F
G
H
I
J
K
L
percentage of clones within tissue
var types

Figure S1. Frequency of cloning of individual *var* types within the organs of Malawian paediatric malaria patients PM 30 (A-D), PM 32 (E-H) and PM 55 (I-L). *Var* types are listed in order of decreasing frequency of cloning within each tissue and are identified by accession number. The dashed line indicates 33% frequency of cloning, the cut-off point for dominance of 1-2 *var* types.
